# Motion of water monomers reveals a kinetic barrier to ice nucleation on graphene

**DOI:** 10.1038/s41467-021-23226-5

**Published:** 2021-05-25

**Authors:** Anton Tamtögl, Emanuel Bahn, Marco Sacchi, Jianding Zhu, David J. Ward, Andrew P. Jardine, Stephen J. Jenkins, Peter Fouquet, John Ellis, William Allison

**Affiliations:** 1 Cavendish Laboratory, University of Cambridge, Cambridge, UK; 2 Institute of Experimental Physics, Graz University of Technology, Graz, Austria; 3 Department of Radiation Oncology, Heidelberg University Hospital, Heidelberg, Germany; 4 Yusuf Hamied Department of Chemistry, University of Cambridge, Cambridge, UK; 5 Department of Chemistry, University of Surrey, Guildford, UK; 6 Institut Laue-Langevin, Grenoble, France

**Keywords:** Physical chemistry, Reaction kinetics and dynamics, Surface chemistry, Surfaces, interfaces and thin films, Graphene

## Abstract

The interfacial behaviour of water remains a central question to fields as diverse as protein folding, friction and ice formation. While the properties of water at interfaces differ from those in the bulk, major gaps in our knowledge limit our understanding at the molecular level. Information concerning the microscopic motion of water comes mostly from computation and, on an atomic scale, is largely unexplored by experiment. Here, we provide a detailed insight into the behaviour of water monomers on a graphene surface. The motion displays remarkably strong signatures of cooperative behaviour due to repulsive forces between the monomers, enhancing the monomer lifetime ( ≈ 3 s at 125 K) in a *free-gas* phase that precedes the nucleation of ice islands and, in turn, provides the opportunity for our experiments to be performed. Our results give a molecular perspective on a kinetic barrier to ice nucleation, providing routes to understand and control the processes involved in ice formation.

## Abstract (web summary)

The dynamics of water molecules at interfaces controls natural and artificial processes, but experimental investigations have been challenging. Here the authors investigate water molecules on a graphene surface using helium spin-echo spectroscopy, and reveal a regime where freely mobile molecules undergo strong repulsive mutual interactions which inhibit ice nucleation.

## Introduction

Ice often forms easily on solid surfaces and to understand why that happens, the molecular basis of the water-surface interaction needs to be studied^1,2^. The structure, dynamics and chemical properties of water at interfaces differ from those of bulk water and ice^3–5^. The early stages of ice nucleation involve exceedingly small time and length scales^6^ and while ice nucleation and phase transitions are well understood macroscopically, unravelling the microscopic details presents one of the great challenges in physical sciences with important implications from the chemistry of the Earth’s atmosphere^7^ to physicochemical processes occurring on cosmic dust grains^8^.

It is the motion of water molecules at surfaces that controls these fundamental phenomena in physics, chemistry and biology as well as a diverse range of technological processes^1,9,10^. Wetting, hydrophobicity and ice nucleation are all very widely studied on the macroscopic scale, using routine methods such as contact angle measurements^11–13^. However, more precise measurements, with a molecular level of detail, are much scarcer, despite the fact that an understanding could open up opportunities for the design of advanced materials, by exploiting our ability to tune surfaces at the nanoscale^14^. For example, ice nucleation on surfaces is alone of huge technological relevance to fields as diverse as wind power^11,15^, aviation^12,16^ and telecommunications^11^.

Water is fundamentally challenging to study with atomic resolution. It is difficult to achieve sufficient contrast and resolution with imaging techniques^17^, particularly in order to understand the position of the H atoms and thus the molecular orientation. Electron-based techniques such as low energy electron diffraction (LEED) also scatter weakly from hydrogen and present a severe risk of damage in the form of water dissociation^18,19^. Some structural studies of water have been possible experimentally, but are usually restricted to flat metal surfaces^18–23^ or a few ionic crystals, such as NaCl^17,24^. These studies have revealed the role of short-range attractive forces. Dynamics and low coverage measurements, which could examine the nature of water interactions more generally, are further complicated by fast diffusion rates and the short lifetimes of water monomers. Insight has therefore been mostly limited to that possible with numerical simulations^25,26^, often without any direct experimental validation to support them.

In this paper we report the serendipitous discovery of a regime where freely mobile water can be studied on a Ni(111) supported graphene surface. Using helium spin-echo (HeSE) spectroscopy, the molecular water motion can be studied with a temporal sensitivity over picosecond timescales while the very low-energy He atoms completely exclude any possibility of damage or dissociation of the water. From the correlation measurements we are able to establish that, contrary to expectations, strong repulsive interactions exist between adsorbed water molecules. We attribute these forces to dipolar interactions arising from structural hindrance of water reorientation by the adsorption geometry. The repulsion leads to a kinetic barrier that inhibits the nucleation of solid ice, while extending the surface lifetime of water monomers and simultaneously making our measurements possible.

## Results

**Fig. 1:**
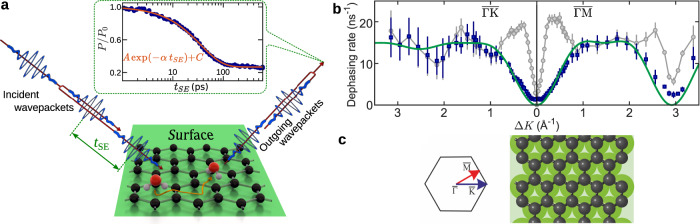
Diffusion of water monomers on graphene. **a** Illustration of the helium spin-echo method: Two wavepackets scatter from the surface with a time difference *t*_SE_, allowing the motion of molecules on the surface to be interrogated through a loss in correlation, measured via polarisation of the beam. The diffusing water molecules are illustrated as white/red spheres for H/O and the atom colours for graphene/Ni are the same as in panel **c**. The inset shows a typical measurement for the diffusion of water on graphene (*T*_*S*_ = 125 K, Δ*K* = 0.2 Å^−1^). The reduction in surface correlation with increasing spin-echo time follows a single-exponential decay (solid line), characterised by the dephasing rate, *α*. **b** The momentum transfer dependence of the dephasing rate, *α*(Δ*K*), at *T*_*S*_ = 125 K from which the mechanism for diffusion follows. Blue data points show single particle, or incoherent *α*(Δ*K*), deduced from the coherent scattering data (grey points, see text). The error bars correspond to the confidence bounds (1*σ*) upon determination of *α* from the measurements (see text). An analytical model (green curve) shows the expected behaviour for jumps between the centres of the graphene hexagons. **c** Structural model for the graphene lattice (grey spheres) on the Ni(111) substrate (green) with the principle symmetry directions ($\overline{{{\Gamma }}\,{{\mbox{M}}}\,}$/$\overline{{{\Gamma }}\,{{\mbox{K}}}\,}$) of the Brillouin zone.

We use the HeSE technique, illustrated in Fig. 1a, to measure surface correlations in the water monomer motion (see “Methods”). HeSE uses wavepacket splitting and recombination to give temporal sensitivity over picosecond timescales^27^, resulting in data of the form shown in the inset of Fig. 1a. The dephasing rates obtained from these correlation measurements are further analysed to provide the signatures of the molecular motion as shown in Fig. 1b and further described in “Diffusion and dynamics of water monomers on graphene”.

### Water adsorption and ice formation on graphene

**Fig. 2:**
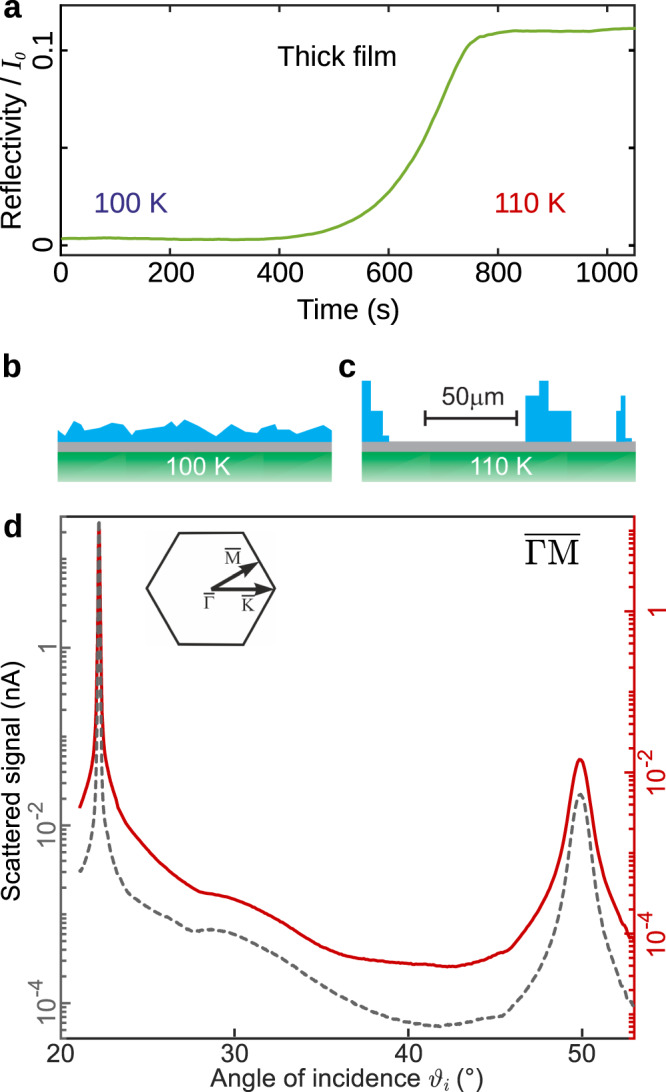
De-wetting of a thick film and island formation. **a** A thick water film prepared at 100 K has low helium reflectivity, indicating a rough surface (time < 200 s). The temperature is increased to 110 K, between 100 and 300 s, and the reflectivity rises later, as the graphene substrate is revealed (time > 600 s)—see main text. **b** and **c** are cartoons indicating the morphology of the surface at 100 and 110 K respectively, with water in blue and the graphene/Ni substrate in grey/green. In both cases it is probably amorphous solid water that forms. At 110 K de-wetting of the surface occurs to reveal pristine graphene. The scale bar gives a qualitative indication of the island separation (see Supplementary Note 1). **d** De-wetting is confirmed as helium diffraction from the graphene film, before water adsorption (grey dashed curve), is identical to that from the thick water film after heating to 110 K (red curve). Both curves are measured at 110 K with an incident beam energy, *E*_*i*_ = 8 meV, plotted with different ordinate limits on the left- and right-hand side. The hexagon shows the principle symmetry directions of the surface Brillouin zone.

In order to identify the range of conditions where individual water molecules are mobile, we carried out extensive adsorption and desorption measurements on the graphene/Ni(111) surface. The substrate was prepared in ultra-high vacuum (UHV) conditions and graphene was grown using established methods^28^ (see “Methods” and Supplementary Methods). Growing a thick film of water at 100 K results in a very low helium reflectivity, which is typical of disordered structures^29–31^ as confirmed by the lack of any helium diffraction. The data indicate an amorphous solid water layer covering the entire surface. Heating the surface slightly leads to a significant change. Figure 2a shows how the reflectivity increases over a period of minutes at 110 K. We can rule out desorption as direct measurements show, that desorption is negligible at this temperature (see Supplementary Note 2). Simultaneously, diffraction peaks emerge at the positions of a graphene lattice, as shown by the red curve in Fig. 2d. The relative diffraction intensities are identical to the pristine graphene surface (grey dashed curve), which would not be the case for a crystalline ice overlayer^32^. Ice I_h_ and ice I_c_ also have too large a lattice spacing^33^ to give rise to this periodicity, even for the spacing in the recently discovered square ice^33,34^. The diffraction pattern indicates that large areas of graphene are exposed, alongside localised areas with multi-layer ice islands on the surface. Thus, we conclude that the deposited water has migrated to form isolated islands of amorphous ice, as illustrated in Fig. 2c. Such behaviour is consistent with the strongly hydrophobic behaviour previously seen on pristine graphene^13,35,36^ and a similar behaviour has been observed for water on other metal-supported graphene systems^33,37^. The formation of islands provides the first indication that in this regime, water molecules must be able to diffuse freely over the graphene surface.

**Fig. 3:**
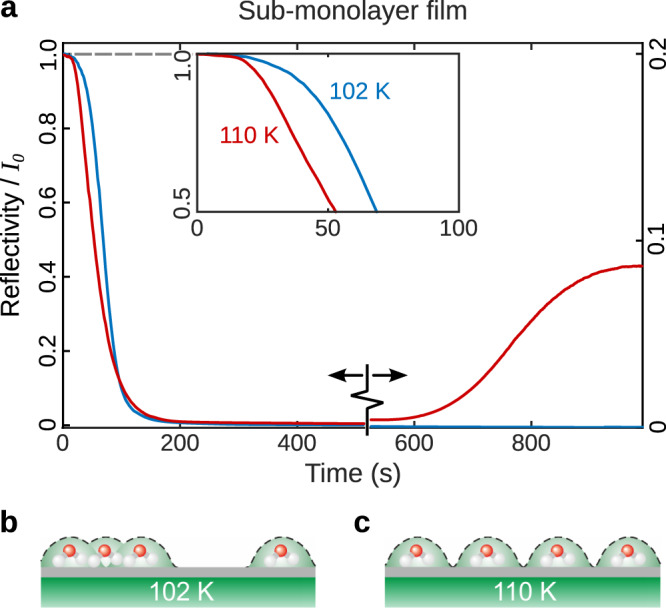
Molecular adsorption in the sub-monolayer regime. **a** Helium reflectivity from a sub-monolayer film, shown as a function of time during constant deposition of water molecules. The reflectivity is a measure of the fraction of the original, pristine surface that is exposed. At low coverages (time < 100 s), the intensity drops as individual water molecules obscure the underlying graphene. The blue curve shows adsorption at 102 K, a temperature where the morphology of thick film measurements (Fig. 2) suggest the molecules are immobile on the time scale of the experiment. **b** Cartoon illustrating the random distribution of immobile H_2_O molecules (white and red spheres) on the graphene/Ni substrate in grey/green. The reflectivity falls rapidly due to the large scattering cross-section of each molecule (dotted lines) despite considerable overlap, even at a fraction of a monolayer. The reflectivity remains low as the thick film, shown in Fig. 2b, is formed. The red curve in panel **a** shows the same rate of adsorption at 110 K, where the reflectivity drops even more quickly (time < 100 s). The faster drop is due to isolated water molecules obscuring a greater area of the graphene surface, as shown in **c**. We attribute the difference to an increased mobility compared to the curve at 102 K, allowing the molecules to achieve a lower energy configuration. It is the first indication that repulsive interactions must keep the water molecules separated. At longer times and higher coverage (time > 600 s), the mobile molecules eventually nucleate ice structures, as discussed in the main text. The surface de-wets, allowing scattering from the underlying graphene to re-emerge, exactly as shown in Fig. 2c.

The observation of de-wetting and island formation is supported by helium reflectivity measurements at very low coverage. Figure 3a compares the behaviour at a surface temperature of 102 K (blue curve), where the molecules are immobile and at 110 K (red curve), where there is some mobility. In both cases, the reflectivity falls sharply when water molecules begin to adsorb because of their large cross-section for diffuse scattering. At 102 K (blue curve), the reflectivity drops to near zero, and remains there throughout the experiment. At 110 K (red curve), the initial drop is even more rapid, despite the same rate of water uptake onto the surface. The observation can be understood if water molecules stay further apart at 110 K than 102 K, as shown schematically in Fig. 3b, c, where the large scattering cross-section of each molecule is indicated by the dotted lines. Regularly spaced molecules at 110 K reduce the overall cross-section overlap, and thus result in the faster reduction in reflectivity with coverage. The additional spacing must arise from water mobility at 110 K, which we discuss below. After further exposure at 110 K, the reflectivity recovers due to the nucleation of islands, at a rate much slower than the molecular diffusion^30,38^. Water molecules desorb at higher temperatures, but up to approximately 130 K it is possible to maintain a constant coverage by applying an over-pressure of water. Under these equilibrium conditions, water monomers diffuse continuously between islands of ice, producing an elusive "free-gas” of monomers, which allows us to study the interactions between isolated molecules prior to ice formation. Differences that might be expected in terms of the film growth with respect to different metal substrates and bulk graphite^5,30,31,39^ are further outlined in the Supplementary Discussion.

### Diffusion and dynamics of water monomers on graphene

In the window of dynamical equilibrium between 113 and 130 K, HeSE experiments were performed to make detailed measurements of water molecular motion. Temporal correlation functions of the form illustrated in the top inset of Fig. 1a describe the dephasing in the scattering due to the motion of water over picosecond timescales and on reciprocal space length scales between 2 and 200 Å. Each measurement can be described by a single-exponential decay function, $A\exp (-\alpha \ {t}_{{{\mathrm{{SE}}}}})+C$, as illustrated by the red line, to obtain the rate of dephasing, *α*. (Uncertainties are the corresponding confidence bounds (1*σ*) of the single-exponential fit.) The variation of *α* in reciprocal space, i.e. as a function of the surface parallel scattering momentum transfer Δ*K* = ∣Δ**K**∣, provides a signature of both the mechanism and rate of the molecular motion.

The grey points in Fig. 1b show the variation of *α*(Δ*K*) for water motion at 125 K along two directions in reciprocal space, at a relative coverage of 0.07 monolayers (ML) (see Supplementary Note 1). Helium atoms scatter coherently and the effects of correlations due to long-range forces between the monomers play a role in the scattering, especially at values of Δ*K* < 1 Å. We discuss the full picture later but first we identify the energy-landscape for motion by removing the effects of pair-correlations among the adsorbates using established methods^40–42^. We use an approximate form for the scattering form factor^42^ and estimates of the quasi-elastic structure factor (see Supplementary Note 4) to obtain the result for incoherent scattering and the corresponding single-particle dephasing rate in Fig. 1b (blue points).

The single-particle dephasing rate (blue points) is periodic in Δ*K* rising from the origin and returning to *α* = 0 at about Δ*K* = 2.9 Å^−1^ in the $\overline{{{\Gamma }}\,{{\mbox{M}}}\,}$ direction. The periodicity indicates that motion takes place by a jump mechanism and the data are well described by a simple model for jump diffusion^27,43^,

1 $$\alpha ({{\Delta }}K)=\frac{2}{\tau }\ \mathop{\sum}\limits_{m}{p}_{m}\cdot {\sin }^{2}\left(\frac{{{\Delta }}{{{\bf{K}}}}\cdot {{{{\bf{j}}}}}_{m}}{2}\right),$$

where the particle rests for a time *τ* within an adsorption site on the corrugated surface, before moving instantaneously to another equivalent adsorption site in a neighbouring unit cell along the vector **j**_*m*_, with probability *p*_*m*_.

The analytic form is shown as a green curve in Fig. 1b and corresponds to jumps on the hexagonal graphene lattice (Fig. 1b), where the jump length is equal to the lattice constant and multiples thereof. The curve includes jumps to nearest, next-nearest and second-nearest neighbours, giving a residence time of *τ* = (65 ± 3) ps and a relative jump contributions of *p*_*n*_ = 63%, *p*_*nn*_ = 20%, and *p*_*nnn*_ = 17%, respectively. Importantly, a jump model can only describe the experimental data if the water molecule is adsorbed in the centre of the hexagons formed by the carbon rings. Jumps with other adsorption geometries would either give rise to a different dependence upon Δ*K* or to the appearance of multiple exponential decays in the data^44^. Hence we can unambiguously determine the adsorption site of water on graphene. These findings are in good agreement with our van der Waals (vdW) corrected density functional theory (DFT) calculations of H_2_O on free-standing graphene (see “DFT calculations” and Supplementary Note 3) and with reported angle-resolved photo-electron spectroscopy of H_2_O on graphene/Ni(111) which have been interpreted in terms of a preferential adsorption on either hollow or bridge sites^45^.

**Fig. 4:**
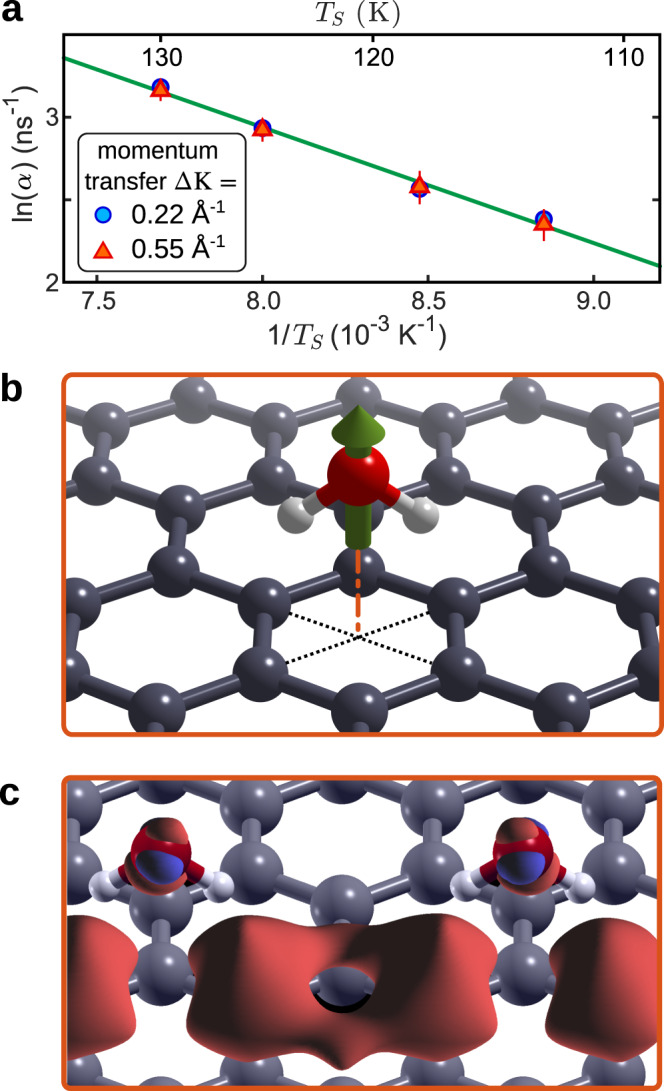
Temperature dependence and theoretical results for water adsorbed on graphene. **a** The temperature dependence of *α* (error bars correspond to 1*σ* confidence bounds) can be used to determine the activation energy for diffusion of water on graphene based on the slope of a linear fit (green solid line). A constant surface coverage of 0.07 ML, corresponding to a reflectivity attenuation factor of 4, was maintained at all temperatures by adjusting the H_2_O over-pressure applied. **b** Adsorption geometry of water (H/O atoms as white/red spheres) on graphene (grey spheres) with the green arrow illustrating the direction of the net dipole of the water molecule. **c** Charge density difference for two water molecules adsorbed on graphene (red/blue isosurfaces correspond to ±0.0025e/Å^3^) illustrating the dipole moment. The dipole moment of a water monomer on graphene is 6.4 × 10^−30^ Cm = 1.9 D, which is slightly larger than for an isolated water molecule.

An accurate tracer diffusion coefficient, *D*, for two-dimensional water motion can be calculated from the residence time, *τ*, using,

2 $$D=\frac{1}{4\tau }{\langle l\rangle }^{2},$$

where 〈*l*〉 = 3.3 Å is the average jump length. Based on the result for single particle motion, we obtain a diffusion constant *D* = (4.1 ± 0.2)  × 10^−10^ m^2^/s at 125 K. An activation energy, *E*_a_, for motion on a particular length scale can be obtained from temperature-dependent measurements using Arrhenius’ law,

3 $$\alpha ={\alpha }_{0}\cdot \exp \left\{-{E}_{{{\mathrm{a}}}}/\left({k}_{{{{\rm{B}}}}}\cdot {T}_{{{\mathrm{S}}}}\right)\right\},$$

where *α*_0_ is a pre-exponential factor relating to the jump attempt frequency, *k*_B_ is the Boltzmann constant, and *T*_S_ is the surface temperature. Figure 4a plots ${{{\mathrm{ln}}}}\,(\alpha)$ versus 1/*T*_S_ and shows a clear linear dependence, giving *E*_a_ = (60 ± 4) meV and *α*_0_ = (5 ± 1) ps^−1^. (Obtained from a weighted linear fit with confidence bounds of 1*σ*.) There is very little difference between the values obtained at the two different momentum transfers shown in Fig. 4a. Our diffusion rates are significantly lower than those from recent molecular dynamics simulations^25,26^, which show very fast diffusion of water droplets and estimated a diffusion coefficient of 6 × 10^−9^ m^2^/s for single water molecules at 100 K^46^. We note that those calculations were performed on free-standing graphene while our measurements are on Ni(111) supported graphene and in particular, the ripples giving rise to the ultra-fast droplet diffusion^26^ are suppressed by the substrate^28^. We also note that our measurements on graphene indicate a higher diffusion and hopping rate than experimental values for other substrates^24,43^ or in nano-confinement^47^ and that the interfacial motion of water is many orders of magnitude faster than bulk diffusion in amorphous solid water^48^ (see Supplementary Table 1 and Supplementary Discussion).

### Intermolecular forces and kinetic barrier to ice nucleation

We now turn to the interactions between water molecules, which are encoded in the differences between the coherent and incoherent rates in Fig. 1b (blue and grey data points). A series of kinetic Monte Carlo (KMC) simulations were performed to determine the nature of these forces. The hexagonal hopping model described earlier was combined with an interaction potential, *V*_pp_, between water molecules, of the form

4 $${V}_{{{\mathrm{{pp}}}}}=\pm\! \frac{B}{{r}^{3}}=\pm\! \frac{{p}^{2}}{4\pi {\epsilon }_{0}{r}^{3}},$$

which represents a pairwise dipole–dipole interaction, where *p* is the effective value of the dipole moment and *r* is the distance separating the two dipoles. Allowing for either positive or negative prefactors provides the ability to explore both repulsive and attractive interactions. Using trajectories from the KMC simulation, simulated coherence functions and dephasing rates were obtained to compare with the Δ*K* resolved experimental data, while also adjusting the simulation to reproduce the temperature dependent measurements.

**Fig. 5:**
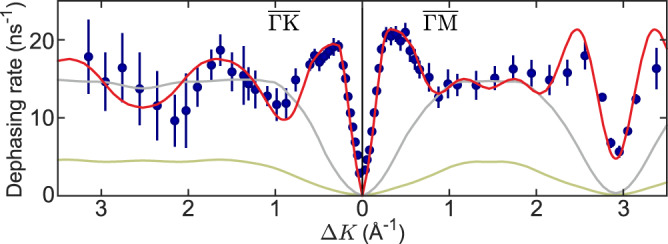
Evidence for repulsive interactions in the diffusion process. Experimental dephasing rates for coherent scattering (blue dots, with the error bars correspnding to the 1*σ* confidence bounds) compared with kinetic Monte Carlo simulations (solid curves). The simulations add a force (Eq. (4)) to the hopping model derived earlier. The experimental data are described well by repulsive dipole forces (red curve) but models using attractive forces (green curve) or no forces (grey curve) cannot reproduce the data. Note that the model without forces (grey curve) is, as expected, similar to the analytic curve for incoherent scattering shown in Fig. 1.

Figure 5 compares the experimental data with KMC simulation results. The red line shows the case of repulsive interactions, where the dipole moment of each molecule has been adjusted to best fit the experimental data, giving a value of *p* = 1.8 ± 0.2 D. There is excellent agreement with the measurements, particularly around the steep rise at 0.5 Å^−1^, a characteristic feature of adsorbate interactions, and around the minimum at 2.9 Å^−1^ in the $\overline{{{\Gamma }}}\overline{{{{\rm{M}}}}}$ direction, where the same phenomenon is seen due to periodicity. The magnitude of the dipole moment obtained from the experiment is in good agreement with the value of *p* = 1.9 D obtained from our DFT calculations. Attractive interactions, by contrast, cannot describe the data. The green curve in Fig. 5 replaces repulsion with attraction of the same strength. Attractive forces suppress the jump rate and do not reproduce the correct dependence on Δ*K*. The reduction in rate is progressive as the magnitude of the forces increases, as might be expected. When there are no interactions (Fig. 5, grey line) the particles move independently and we obtain the same form as the analytical model for hopping in Fig. 1b.

Together, these observations provide conclusive confirmation of significant long-range repulsive interactions between water monomers. These interactions act to keep the water monomers apart, thus suppressing nucleation and increasing both monomer lifetime and mobility. It is well known that short range attractive forces and hydrogen bonding play a major role in the clustering of water. We therefore conclude that there must be an intermediate barrier between the long-range repulsive regime and the short range attractive one. This intermediate barrier must be overcome for ice nucleation to occur. Note that this barrier is in addition to the activation energy required to hop between sites and the energy required to re-orient the molecules relative to each other. The additional energy barrier we observe represents a fundamental change in our understanding of ice formation on surfaces, where the intermolecular forces have been assumed to be exclusively attractive.

Questions about the origin and form of the barrier immediately arise and how they relate to the dipole moment of the water molecules^49^. Our DFT calculations, shown in Fig. 4b-c, indicate that water molecules all adsorb with the same orientation, with a dipole moment slightly larger than for an isolated water molecule. The alignment of individual dipoles perpendicular to the surface plane, leads to strong repulsive interactions and is the likely origin of the forces we observe. In order for a water cluster to nucleate, molecules must first come into close proximity and must then re-orient to adopt a hydrogen bonded configuration. Both of these steps have separate energy barriers, such that in combination they strongly inhibit the overall process. Repulsive interactions between adsorbates occur widely at surfaces and can limit the density of adsorbed species as well as defining the adsorbate structure (see, for example refs. ^50–55^ and the Supplementary Discussion). In the present work, a kinetic barrier arising from repulsive forces of adequate range is a different mechanism that provides insight into the inhibition of ice nucleation.

Cluster nucleation is closely related to the process of water molecule attachment to existing islands—although once ice has nucleated, dipole repulsion from the island is reduced, such that only the re-orientation barrier is relevant. The lifetime of each water molecule on the surface before it sticks to an island can be estimated from the ratio of the equilibrium coverage, 0.07 ML = 8 × 10^17^ m^−2^, and the adsorption rate, 2.5 × 10^17^ m^−2^ s^−1^, giving roughly 3 s at 125 K (see Supplementary Note 1). The very long lifetime compared to the residence time in any given adsorption site reflects the difficulty in overcoming any one of these barriers. We also suggest that desorption of water molecules in our experiments is only likely to happen from the surface of water islands since our experimentally determined desorption energy of (520 ± 20) meV (see Supplementary Note 2) is close to the sublimation enthalpy from ice^56,57^.

## Discussion

Finally, we can consider our results in the broader context of ice formation. Our measurements on water monomers were only possible as we discovered a regime where, within a small temperature window, individual water molecules diffuse in dynamic equilibrium with islands of ice and where the molecules have lifetimes long enough to apply HeSE measurements. The lack of other data on water monomer dynamics means it is difficult for us to make completely definitive statements about the generality of our observations. However, dipole formation occurs widely upon adsorption^58^ and whenever those dipoles are prevented from re-orienting during diffusion, dipole-driven inter-adsorbate repulsion is viable. Such interactions have been observed between hydrocarbons^52^ and between alkali metals^59^ on metal substrates, but to our knowledge a powerful suppression of ice nucleation arising from strong intermolecular repulsion between water molecules has not been reported up to now. Attractive forces and hydrogen bonding, which are dominant after the onset of ice growth at higher coverage, have always been assumed. It represents an important step in unravelling the unique behaviour of ice and the complex relationships between adsorption, jump diffusion and long-range intermolecular interactions.

Our findings also suggest broadly applicable strategies for further suppressing or otherwise controlling the ice nucleation process, by enhancing the dipole formed during adsorption. Such an effect could be achieved by, for example, using surface treatments leading to greater electron transfer, or in the case of graphene by altering the supporting substrate. In these respects, the hydrophobic character of the graphene substrate^35,36,60^ and particularly the adsorption geometry play important roles, but it seems reasonable to expect that the dipolar effect could apply much more generally in water adsorption at surfaces.

## Methods

### Experiment and sample preparation

Gaining direct images of water on non-metallic surfaces remains challenging because of the weak interaction of single water molecules with those substrates. For example, on graphene, water has previously only been visualised when subsurface, due to its dynamic nature^61,62^. Compared with other techniques, He atom scattering has the advantage of being the most delicate surface-probing technique and is sensitive to H atoms in the top layer^63–66^. All measurements have therefore been performed using the Cambridge helium-3 spin-echo facility (HeSE)^27,67,68^. The schematic principle of He spin-echo is illustrated in Fig. 1a: A polarised He beam, illustrated by the blue wavepacket, is split into two components which are separated in time by *t*_SE_. After scattering from the surface, the separated wavepackets are recombined. If the surface changes between scattering of the two parts of the wavepacket, a loss of polarisation is observed in the detected beam, which is directly related to the change in surface correlation and in the case of surface diffusion, usually follows an exponentially decaying form (see refs. ^27,67,68^ for more information).

The preparation of a single graphene layer on Ni(111) is described in ref. ^28^ and the Supplementary Methods. Water was dosed onto graphene with a microcapillary array beam doser which was brought close to the surface. During H_2_O dosing, the partial pressure of water in the scattering chamber was maintained using an automatic leak valve, and the helium reflectivity monitored. During dynamics measurements, dosing was adjusted to achieve a certain attenuation of the helium reflectivity, which corresponded to a particular coverage. Reflectivity was regularly checked to ensure equilibrium was maintained during individual experiments and between measurements under the same conditions, to ensure reproducibility.

A microcapillary array beam doser was used for depositing water on the nickel surface. The doser was moved to 5 cm distance from the sample to reduce the water load in the scattering chamber and the dose was estimated from the water pressure in the chamber and the enhancement factor, which is known from previous works (see Supplementary Note 1). Water was supplied to the doser from a baked stainless steel tube filled with de-ionised water, using the vapour pressure over the liquid phase at room temperature. The water was purified using a process of several freeze–pump–thaw cycles, where the water inside the tube was frozen and the gas phase above the frozen ice was pumped away. Several repeated cycles were performed until a quadrupole mass spectrometer in the scattering chamber only showed pure water. The water was re-purified prior to every series of adsorption, diffraction, or He spin-echo measurements and regular mass spectrometer scans were performed throughout the measurements to exclude the possibility of contamination.

### DFT calculations

We performed calculations using CASTEP^69^, a plane wave periodic boundary condition code. The Perdew Burke Ernzerhof^70^ exchange correlation functional, with the dispersion force corrections developed by Tkatchenko and Scheffler (TS method)^71^, was employed for all the calculations presented in this work. The plane wave basis set was truncated to a kinetic energy cutoff of 360 eV. The calculations are performed on a (6 × 6) graphene cell, carbon atoms are fixed, *k*-point sampling has been done with a (2 × 2 × 1) MP grid^72^. A vacuum layer of 15 Å was imposed above the graphene surface in order to avoid spurious interactions with the periodically repeated supercells. All the calculations use Vanderbilt Ultrasoft Pseudopotentials^73^ and the *x*, *y* coordinates of the O atoms are fixed. The electron energy was converged up to a tolerance of 1 × 10^−8^ eV while the force tolerance for the geometrical optimisations was 0.05 eV/Å.

### KMC simulations

KMC simulations employing a modified form of the Metropolis algorithm were used to provide insight into the mechanism of adsorbate interactions during diffusion^43,74,75^. Water molecules move on a hexagonal lattice with jumps up to third nearest neighbour sites. A periodic (60 × 40) grid was used, where H_2_O molecules were were initially located on grid sites at random. The potential energy for a molecule at each site in the grid was calculated for the initial configuration, taking into account repulsive/attractive inter-adsorbate interactions using a pairwise dipole–dipole potential of the form described in Eq. (4) (main text).

Each MC step consists of choosing a water molecule at random which may then hop to one of its neighbouring sites, with specific probabilities for jumps to first, second and third nearest neighbours. Provided that the water molecule is not blocked from entering the new site by another molecule, the probabilities are weighted by the difference in the potential of the molecule at the two sites. If several new sites with lower potential energy exist, one of them is chosen at random and the molecule is moved into the new site.

### Reporting summary

Further information on research design is available in the Nature Research Reporting Summary linked to this article.

## Supplementary information

Supplementary Information

Reporting Summary

## Data Availability

The datasets generated and analysed during the current study are available from the University of Cambridge Apollo repository, with the identifier 10.17863/CAM.55076 (ref. ^76^).

## Code availability

The code for the kinetic Monte Carlo simulations is available from 10.5281/zenodo.3240428 under the GNU/GPL-3.0 license.
