# Author Correction: Motion of water monomers reveals a kinetic barrier to ice nucleation on graphene

**DOI:** 10.1038/s41467-021-27211-w

**Published:** 2021-11-18

**Authors:** Anton Tamtögl, Emanuel Bahn, Marco Sacchi, Jianding Zhu, David J. Ward, Andrew P. Jardine, Stephen J. Jenkins, Peter Fouquet, John Ellis, William Allison

**Affiliations:** 1 Cavendish Laboratory, University of Cambridge, Cambridge, UK; 2 Institute of Experimental Physics, Graz University of Technology, Graz, Austria; 3 Department of Radiation Oncology, Heidelberg University Hospital, Heidelberg, Germany; 4 Yusuf Hamied Department of Chemistry, University of Cambridge, Cambridge, UK; 5 Department of Chemistry, University of Surrey, Guildford, UK; 6 Institut Laue-Langevin, Grenoble, France

**Keywords:** Physical chemistry, Reaction kinetics and dynamics, Surface chemistry, Surfaces, interfaces and thin films, Graphene

Correction to: *Nature Communications* 10.1038/s41467-021-23226-5, published online 25 May 2021.

The original version of this Article contained an error in ref. ^14^, which was incorrectly given as: “Akhtar, N. et al. Pillars or pancakes? Self-cleaning surfaces without coating. *Nano Lett*. **18**, 7509–7514 (2018).” The correct form of ref. ^14^ is: “Akhtar, N., Anemone, G., Farias, D. & Holst, B. Fluorinated graphene provides long lasting ice inhibition in high humidity. *Carbon* **141**, 451–456 (2019).” This has been corrected in the PDF and HTML versions of the Article.

