# Supplementary material for: Motion of water monomers reveals a kinetic barrier to ice nucleation on graphene
Source: Nat Commun. 2021 May 25;12:3120. doi: 10.1038/s41467-021-23226-5 (PMC8149658; doi:10.1038/s41467-021-23226-5)
Supplement: Supplementary file 1 — Supplementary Information [file 41467_2021_23226_MOESM1_ESM.pdf]

Supplementary Information

Motion of water monomers reveals a kinetic barrier to ice nucleation on graphene.

Tamtögl *et al.*

Supplementary Methods

The growth and characterisation of the graphene layer on a Ni(111) surface has been published elsewhere[1]. In short, the nickel (111) single crystal used in the study was mounted onto a sample holder and can be heated radiatively by a filament on the back of the crystal, or cooled to 100 K using liquid nitrogen. Prior to the measurements, the Ni surface was cleaned by repeated cycles of Ar^+ sputtering and annealing at 870 K. A monolayer of graphene on Ni(111) was grown by dosing ethene (C_2H_4) while holding the crystal at 730 K for several hours.

The sample temperature was measured using a chromel-alumel thermocouple. While absolute temperatures can be determined to an accuracy of ± 5 K, relative temperature values were determined with an accuracy of ± 0.1 K, which was also confirmed by the reproducibility of the adsorption and dynamics measurements.

Water adsorption and desorption processes were studied during dosing with a precise water pressure control obtained by a motorised leak valve attached to the dosing supply. The leak valve itself was regulated by a feedback control system in order to maintain a constant pressure. Adsorption was measured at sample temperatures of 100, 110, 125, 130 and 150 K at a typical dosing pressure at the surface of $(2 - 20) \cdot 10^{-9}$ mbar.

The helium reflectivity, at 100 K as shown in Figure 3 of the main article decreases continuously and remains low when the dose is stopped. Such a behaviour is typical of disordered structures forming, e.g. the growth of an amorphous layer. The formation of amorphous ice layers on surfaces, commonly referred to as amorphous solid water (ASW) has been observed since the 1960s[2]. For example, recent isothermal desorption measurements of water on highly oriented pyrolytic graphite (HOPG) at 100 K, showed a glass transition accompanied by a change in desorption rate and a growth of 3D water islands, rather than a wetting of the graphite surface[3]. At 110 K and 125 K, the helium reflectivity also decays during deposition, but reaches saturation. Based on the fact that exactly the same diffraction pattern is observed as from clean graphene, the deposition has been interpreted as forming separated islands of ice, leaving areas of bare graphene in between (see main text).

For sample temperatures above 120 K, when the applied pressure is reduced after dosing the helium reflectivity recovers very quickly. The system is thus in a dynamic equilibrium where small changes in the pressure immediately affect the coverage and hence the reflectivity. While with increasing overpressure the coverage increases, with increasing surface temperature the dynamic equilibrium is also reached faster. Within the available temperature range – where we could observe diffusion and where we are able to obtain a constant coverage by applying an overpressure – it was found that measurements at 125 K provided the best trade-off in order to clearly see dynamics and maintain a constant coverage. Dephasing rate measurements at 125 K are reported in the main text (Figure 1b), but further measurements at 130 K, indicate that within experimental uncertainties the measurements have the same variation with scattering momentum transfer, ΔK, and thus the same mechanism of motion at different temperatures. Finally, at even higher surface temperatures, i. e. around 150 K and above, negligible adsorption is observed, even when pressures of up to $1.5 \cdot 10^{-7}$ mbar were applied.

Supplementary Note 1: Details about coverage calibration and the monomer lifetime

All measurements were performed at the same coverage of 0.07 ML, which we define using a particular value of reflectivity, $I/I_0 = 0.25$. The reflectivity is adjusted, at each temperature, by varying the overpressure of water vapour from the capillary doser (see Methods in the main text). We estimate the value of the coverage when $I/I_0 = 0.25$ using the measured dynamical data (Figure 5, main paper) and, in particular, the shape and position of the features arising from the pairwise inter-adsorbate forces. The method provides an absolute measure of coverage since the prominent minimum in the data ($|\Delta \mathbf{K}| \approx 0.8\,\text{Å}^{-1}$) corresponds to a quasi-hexagonal overlayer with a spacing that defines the coverage. Varying the coverage in the kinetic Monte-Carlo calculations leads to the red curve in Figure 5 of the main paper, which corresponds to a coverage of (0.07 ± 0.02) ML (where 1 ML is defined as one water molecule per adsorption site).

A second method of coverage calibration is less direct but it serves to confirm the internal consistency in our measurements and their interpretation. Here, we use the dosing rate to calculate the coverage, assuming a constant sticking coefficient of unity (see Refs.[4–6], with monolayer coverage corresponding to $n = 0.115$ molecules/Å^2,[7] which is close to the density of an ice I_h overlayer[5]). The rate of impingement is calculated from the measured partial-pressure of water in the chamber and the known enhancement generated by the microcapillary array[8]. Using that approach we can show that upon water adsorption at 100 K, the reflectivity follows a model for random adsorption (stick and sit, see ref.[9]), with remarkable agreement over two-orders of magnitude. Hence it confirms our assumption that the water monomers are static at 100 K. Also, the method allows us to estimate a scattering cross section for isolated water monomers of $\Sigma = (120 \pm 20)\,\text{Å}^2$, consistent with the coverage calibration derived from the dynamic measurements. Here, most of the quoted tolerance arises from uncertainties in the doser calibration and doser position relative to the sample. The determined cross section is in good agreement with other water cross sections in the literature on similar systems (e.g. $\Sigma = 130\,\text{Å}^2$ in Ref[10]).

The fraction of the surface covered by ice islands cannot be estimated from the scattering data since the diffraction patterns with and without water adsorption are essentially identical (Figure 2d, main paper). It follows that the area of the surface covered by ice is extremely small. We can estimate the separation of ice islands from the r.m.s. distance travelled by an H_2O monomer using known conditions for dosing and the measured diffusion constant. At 125 K the impinging flux of molecules was $2.5 \cdot 10^{17}\,\text{m}^{-2}\text{s}^{-1}$ and the corresponding rate of adsorption is 0.022 monolayer per second. Taking the coverage of water monomers, at equilibrium, to be 0.07 ML we obtain 3.4 s as the average lifetime of an H_2O monomer, after adsorption and before being bound to an island. From the dynamics measurements in the main part of the manuscript we know that the hopping rate at 125 K is $1.5 \cdot 10^{10}\ \text{s}^{-1}$. The mean jump length during diffusion is 3.3 Å and it follows that a monomer travels about 70 μm before sticking to an island. The island separation must therefore be on a similar scale, which leads to the approximate scale-bar indicated in Figure 2c (main paper).

Supplementary Note 2: Desorption measurements

Several groups have conducted thermal desorption spectroscopy (TDS) measurements of water on the (0001) basal plane of graphite. Consistently, a single desorption peak was observed that corresponds to a desorption energy in the range of 0.4 to 0.5 eV which is close to the sublimation enthalpy of ice, 0.49 eV[5,6,11]. The observed desorption energy does not change with coverage, indicating the formation of separated islands on the graphene surface[11].

On the surfaces of graphene/Ni(111) and of graphene/Ir(111),

TDS spectra reveal pseudo-zeroth order desorption and desorption energies of $(356{\pm}23)$ meV in the first case, and $(585{\pm}31)$ meV in the latter case, respectively, were found [12]. Smith *et al.* report a desorption energy of about 490 meV for graphene/Pt(111), however they noted that the desorption of the water monolayer is complicated by de-wetting upon desorption and only multi-layer water films show zero-order desorption [7].

We have also conducted thermal desorption spectroscopy while monitoring the $m/z = 18$ peak on a mass spectrometer and simultaneously measuring the helium reflectivity. A single desorption peak with a maximum at 163 K coincides with a rapid recovery of the specular signal. The Redhead equation can be applied, in order to estimate the desorption energy E_d. Using $\nu = 9 \cdot 10^{-14}$ s^{-1} according to Ulbricht *et al.* [11] for the peak maximum at (163 ± 5) K at a heating rate $\beta = 0.22$ K $\cdot$ s^{-1}, we obtain a desorption energy of $E_d = (520 \pm 20)$ meV.

Furthermore, we can use the recovery of the helium reflectivity to determine the desorption energy. We exposed the graphene surface to a water overpressure of $2 \cdot 10^{-8}$ mbar and waited until the system was in equilibrium, before turning off the exposure and monitoring the reflectivity recovery. From this we calculated the corresponding surface coverage as a function of time. The surface coverage first rises during exposure and then decays exponentially after exposure has been turned off. The initial desorption rate, which is identical to the exponential decay rate, exhibits an activated temperature dependence. The desorption energy can then be determined from the slope in an Arrhenius plot and gives a value of $E_d = (510 \pm 10)$ meV, in good agreement with the conventional TDS method.

As mentioned in the main text, it suggest that water molecules tend to desorb from the surface of water islands into the vacuum rather than as individual molecules which are adsorbed on the bare graphene surface - with the latter being more likely to diffuse and bind to an island. These findings are further supported by the diffraction measurements stated in the main text and the mentioned de-wetting upon desorption as observed for water on graphene/Pt(111) [7].

Supplementary Note 3

The density functional theory (DFT) approach has been applied a number of times to the adsorption of water on graphene. DFT calculations generally agree that the potential energy surface is rather flat and that the binding energy depends more on the orientation than on the position of the adsorbent. Most calculations predict a preferential water adsorption with the hydrogen atoms pointing downwards (see Figure 4b in the main paper and Supplementary Figure 1a). We obtained an adsorption energy, E_{ads}, of about 250 meV for the global minimum, which is located at the centre of the graphene hexagonal unit cell. The value is in very good agreement with the 183 meV obtained by Li *et al.* [13], while other DFT calculations give slightly smaller adsorption energies [12,14–17]. We also find the water molecule is adsorbed with the orientation usually predicted, i.e. that it has the two OH bonds pointing towards the surface, so that the plane of the molecule is perpendicular to the surface plane itself. A general agreement on an adsorption distance of about 3.3 Å can be observed between our results and previous reports [13,15,18,19].

In addition to calculations on bare graphene, we performed a set of vdW DFT calculations including a Ni substrate (modelled as a five-layers nickel slab with a $(\sqrt{7} \times \sqrt{7})$ surface unit cell) to verify that the Ni substrate does not affect significantly the nature of water interaction with graphene. We found that the water to graphene distance and the orientation of the water monomers are very close to the calculations on the pristine graphene without the Ni substrate, although the absolute adsorption energy with the substrate is slightly different, ≈ 230 meV compared to 250 meV for suspended graphene. Including the substrate necessarily reduces the size of the surface unit cell and, by omitting the Ni, it is possible to increase the unit cell significantly (a 9-fold increase in the number of carbon atoms). The larger supercell allows us to model adsorption at significantly lower coverages, much close to those in the experiment. For these reasons, we used a larger graphene supercell without substrate to investigate the dynamics of water motion at low coverage, as presented in the manuscript.

We estimate the magnitude of the energy barrier to dimer formation by performing a series of DFT calculations for two water monomers (in the same orientation as the isolated monomer) with varying distance. The resulting barrier is about 90 meV, while the binding energy of the dimer is approximately 200 meV. Thus, the barrier to dimer formation is significant and we can conclude that, once a dimer forms, it will rarely dissociate. The dimer exhibits then a total adsorption energy of $595 - 696$ meV (depending if the substrate is frozen or not). These results support also our observation of hysteresis during isobaric cooling/heating and they are consistent with previous calculations of of H_2O clusters adsorbed on graphite where the association energy (including re-orientation, binding and adsorption) with the cluster is in the range of $450 - 500$ meV, while the binding energy of a monomer to the graphene surface is much lower [15,18].

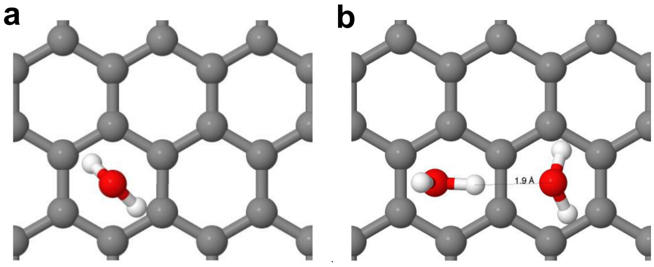

Supplementary Figure 1: **Adsorption geometries of water according to DFT calculations. a** Adsorption geometry for a single H_2O molecule on graphene according to our DFT calculations. **b** Optimised adsorption geometry after the formation of an H_2O dimer on graphene.

In the measured ΔK-range of this study, the dephasing rate α is in the order of 10 ns^{-1} at 125 K. This temperature corresponds to a mean kinetic energy in the order of 10 meV. Since the adsorption energy of an H_2O molecule in an ice cluster is predicted to be in the order of 500 meV [15], while for the adsorption energy of a molecule on the graphene surface, values in the order of $100 - 200$ meV have been calculated, one would expect to observe the diffusion of H_2O on graphene, rather than on the surface of an ice cluster. Together with the adsorption and diffraction results this is another evidence that we are seeing the motion of single water molecules on graphene.

Supplementary Discussion

Water diffusion on other substrates Supplementary Table 1 compares our activation energies and diffusion constants (top row) with water measurements on other substrates in the literature.

In addition, the diffusion of water on graphene has been recently studied by means of molecular dynamics (MD) simulations. Tocci *et al.* predict a substantially lower macroscopic friction coefficient in comparison to adsorption on a hexagonal boron nitride surface [23] and Park *et al.* predicted fast diffusion with a diffusion constant $D = 2.6 \cdot 10^{-8}$ m^2/s [24]. MD simulations of water nanodroplets on freestanding graphene [25] revealed a diffusion con-

Supplementary Table 1: Comparison of experimentally determined diffusion parameters for water monomers on different substrates, including the activation energy, E_a, the diffusion constant, D_0, in Arrhenius pre-exponential form, and the hopping attempt rate, Γ_0.

Substrate	Adsorbate	E_a (meV)	D_0 (m^2s^{-1})	Γ_0 (s^{-1})	T range (K)	Ref.
**Graphene/Ni(111)**	$\mathbf{H_2O}$	**60**	$\mathbf{1.1 \cdot 10^{-7}}$	$\mathbf{4.0 \cdot 10^{12}}$	$\mathbf{113 - 130}$	**present work**
Cu(111)	D_2O	75	$1.8 \cdot 10^{-8}$	$1.8 \cdot 10^{11}$	$23 - 29$	[20]
Bi_2Te_3(111)	H_2O	34	$1.3 \cdot 10^{-8}$	$1.7 \cdot 10^{11}$	$130 - 160$	[21]
NaCl(001)/Ag(111)	D_2O	149	$1.5 \cdot 10^{-8}$	$1.0 \cdot 10^{12}$	$42 - 52$	[22]

stant between $2 \cdot 10^{-7}$ m^2/s and $8.6 \cdot 10^{-7}$ m^2/s depending on the size of the droplet (at 298 K). Both values are way beyond the diffusion constant found in our experiments, yet they are considering the motion of water clusters and droplets at much higher temperatures (room temperature) rather than the diffusion of monomers. The diffusion coefficient for single water molecules on graphene has been estimated to be $6 \cdot 10^{-9}$ m^2/s at a temperature of 100 K by Ma *et al.* [14] which is somewhat closer to the conditions in our experiments. Indeed their value is closer to our result but still one order of magnitude larger. However, all calculations mentioned above were performed on free standing graphene while our measurements are on graphene/Ni(111) where the motion of the ripples which gives rise to the ultra-fast diffusion [25] is suppressed [1].

Compared to the diffusion in (bulk) amorphous ice on the other hand, where for the translational motion D_0 is in the range of $(0.5 - 5) \cdot 10^{-17}$ m^2/s [26], or the diffusion of ASW at the liquid/ice interface with $D \approx 10^{-21}$ m^2/s at 125 K [27], the diffusion of water monomers on graphene with $D = 4.1 \cdot 10^{-10}$ m^2/s at 125 K is incomparably faster. We also note that the mobility on graphene is higher than translational diffusion of water in nanoconfinement [28].

Adsorption on graphene and bulk graphite The water dynamics may be affected by the metal substrate [7] or the presence of the bulk, in the case of graphite [29]. Most previous studies are performed in a different temperature or coverage regime so detailed comparisons are problematic. For example, on graphite [29] there is evidence that ice with a thickness of hundreds of monolayers coexists with regions of bare graphite, similar to our observations. In terms of the adsorption energies, vdW DFT predicts that for supported graphene, about 30% of the vdW interactions between the water and the substrate are transmitted through graphene [30]. Moreover, for graphene on metal substrates where a Moiré superstructures with a periodic height variation of the graphene layer forms, it has been reported that the regions closest to the metal substrate act as nucleation centres [31]. The desorption energies on the other hand are all within a similar energy region, both for water on metal supported graphene as well as for graphite [7,29,32], however, desorption results are typically complicated by de-wetting upon desorption as further discussed in Supplementary Note 2: Desorption measurements

Repulsive forces in adsorbate structure and dynamics On the one hand, inter-adsorbate repulsion occurs widely at surfaces and as mentioned in the main text these can limit the adsorbate density as well as defining the adsorbate structure. A typical example where it is commonly assumed that pairwise repulsion exists are (oriented) CO molecules [33,34]. On the other hand, processes such as thin-film growth and nucleation require attractive interactions and attractive forces are therefore often assumed to dominate the process. Our observations show that repulsion can control the surface motion, creating in the case of water molecules adsorbed on graphene a kinetic barrier to nucleation.

However, the specific signature of repulsive interactions in surface diffusion remains a subject which is typically addressed via indirect approaches [35] while direct experimental evidence about repulsive interactions in adsorbate dynamics has only been addressed in very few cases such as for the diffusion of Na [36]. For example, the repulsion between CO molecules in the high-coverage regime based on information from structural organisation and adsorption sites [33,34], could not be confirmed by surface diffusion measurements in the low- to medium coverage regime [37].

Supplementary Note 4: Details about single particle and collective motion

The trajectories of the molecules versus time resulting from the KMC simulations can be used to calculate the intermediate scattering function (ISF) which is also obtained in the experiment. From the KMC simulation both the coherent and the incoherent ISF can be calculated. The subtle difference between the coherent and incoherent ISF is the averaging procedure. While the coherent ISF is obtained by averaging over all particles, the incoherent ISF is obtained by first calculating the ISF of a single particle followed by averaging over all particles. Details on how to obtain both the coherent and the incoherent ISF can be found elsewhere [38].

$$
\begin{aligned}
I(\Delta K, t)_{coh} &= \frac{1}{N} \sum_{j,k}^{N} \left\langle \mathrm{e}^{\mathrm{i}\Delta \mathbf{K}\left(\mathbf{R}_j(t) - \mathbf{R}_k(0)\right)} \right\rangle , \\
I(\Delta K, t)_{inc} &= \frac{1}{N} \sum_{j}^{N} \left\langle \mathrm{e}^{\mathrm{i}\Delta \mathbf{K}\left(\mathbf{R}_j(t) - \mathbf{R}_j(0)\right)} \right\rangle ,
\end{aligned} \tag{1}
$$

The ISFs obtained from the simulation are then analysed in the same way as the experimental data: The ISF is fitted with a single exponential decay which allows to determine the dephasing rate $\alpha(\Delta K)$ from the simulation in analogy to the curve determined from the experiments. The trajectories from the KMC simulation can be used to calculate both the coherent and the incoherent ISF.

On the other hand, He spin-echo is a coherent scattering method, hence the measurements provide the coherent ISF. As shown for neutron scattering [39] as well as for X-ray photocorrelation spectroscopy [40] one can de-correlate the effect of adsorbate interactions, i.e. obtain the corresponding incoherent $\alpha_{inc}(\Delta K)$ from the measured coherent $\alpha_{coh}(\Delta K)$ [38].
Following the derivation of Sinha and Ross [41], where the interaction forces are considered as a mean field, the scattering function S_s of the non-interacting system becomes:

$$
S_s(\Delta K, \omega) = \frac{\Gamma_s(\Delta K)\, c\,(1-c)}{\pi\left(\Gamma_s(\Delta K)^2 + \omega^2\right)}, \tag{2}
$$

where c is the concentration of dynamic adsorbates, and the quasi-elastic broadening Γ_s follows the well established Chudley-Eliott lineshape (the analytical model as in the main text) [38].

The only region where this approach does not apply is in the

vicinity of the substrate diffraction peaks. Here the structure factor of the substrate becomes important while at the same time the uncertainty of the quasi-elastic amplitude becomes large. As a consequence the blue dots in Figure 1b of the main text show an offset for ΔK close to zero and around the diffraction peaks.

Finally, here we note again that only the implementation of repulsive interactions in the KMC simulation can reproduce the peak-and-dip structure as evident in the experimental data. Supplementary Figure 2a shows both the dephasing rate α (top panel) and the corresponding static structure factor $S(\Delta K)$ (lower panel) as extracted for the KMC simulations along the $\overline{\Gamma\mathrm{K}}$-azimuth. We see that only for repulsive forces ($B > 0$, equation (4) in the main text) there appears a clear peak in $S(\Delta K)$ at the same position, where $\alpha(\Delta K)$ shows a dip, as illustrated by the dash-dotted vertical line.

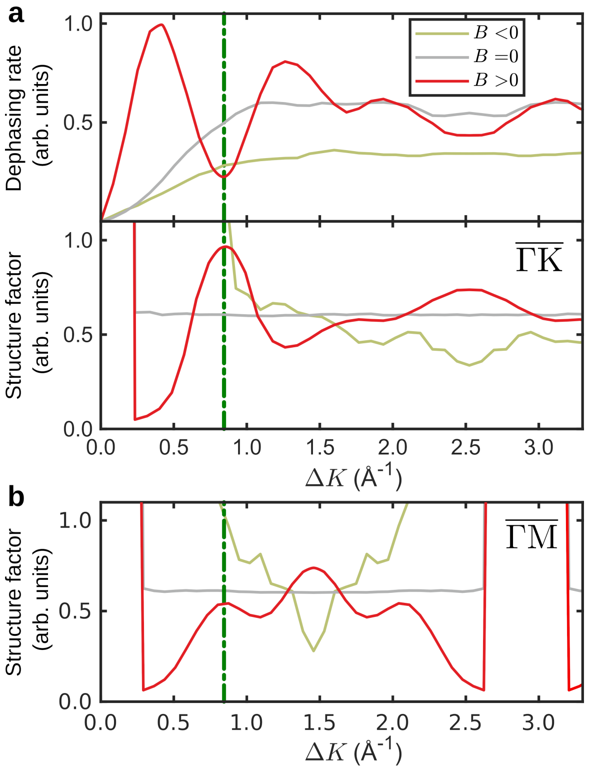

Supplementary Figure 2: **a** Comparison between the dephasing rate α (upper panel) and the corresponding static structure factor $S(\Delta K)$ (lower panel) along the $\overline{\Gamma\mathrm{K}}$-azimuth, as extracted from the KMC simulations at the same conditions (including temperature and water coverage) as for the experiment. Only repulsive interactions ($B > 0$) give rise to a clear peak in $S(\Delta K)$ at the same position where $\alpha(\Delta K)$ shows a dip as illustrated by vertical dash-dotted line. **b** Static structure factor $S(\Delta K)$ along the $\overline{\Gamma\mathrm{M}}$-azimuth.

The repulsive forces between the adsorbates give rise to a deviation of the dephasing rate $\alpha(\Delta K)$ with respect to the analytical expression (equation (1) in the main text). Adsorbates repelling each other prefer a long-range quasi-hexagonal structure leading to a preferred, coverage dependent average distance (see Supplementary Note 1) between the adsorbates and reduced mobility on these length scales[36,42,43]. At the same time, when adsorbates approach each other their mobility increases compared to the non-repelling case. The result is a peak at lower ΔK followed by a dip feature, termed as "de Gennes narrowing" as illustrated by the red line in the top panel of Supplementary Figure 2a. We see that the grey line from the KMC simulation without repulsive interactions follows the same sinusoidal curve as for the analytical expression while the red line - illustrating the case for inter-adsorbate repulsion - exhibits a peak appearing at low ΔK values due to the increased mobility at certain length scales, followed by a dip (vertical dash-dotted line) occuring at the length scale of the quasi-hexagonal arrangement.
The location of this dip corresponds to a peak in the static structure factor[44], as seen in the lower panel of Supplementary Figure 2a and Supplementary Figure 2b. The peak in $S(\Delta K)$ appearing at $\Delta K \approx 0.8\,\text{Å}^{-1}$, designated by the green vertical line, corresponds to an intermolecular distance of about 8 Å in real space. The preferred average distance during the diffusion of water monomers on graphene in the low-coverage regime is thus much larger compared to any spacing observed in X-ray diffraction from amorphous or polycrystalline bulk ice. In the latter case the first peak in $S(\Delta K)$ appears at $1.7\,\text{Å}^{-1}$ and hence at much smaller intermolecular distances in real space[26], as expected.
However, it is important to note that the dynamics measurements in the low coverage regime do not provide us with information about the actual structure of the adsorbate layer. Structural information can instead be taken from diffraction measurements such as Figure 2d in the main text - which in the current scenario are conclusive about the formation of a disorderd film in the high coverage regime. Moreover, the mentioned static structure factor in the case of X-ray diffraction is an isotropically averaged $S(\Delta K)$ due to being from a polycrystalline/amorphous sample[26] meaning that the radial distribution function can be obtained in a straightforward Fourier transform. $S(\Delta K)$ for 2D diffusion in a HeSE experiment, on the other hand, will be direction dependent. It is determined by the azimuthal orientation of the crystal - given by $\mathbf{\Delta K}$ - and provides a measure of the projection along that direction[42] as seen for the $\overline{\Gamma\mathrm{K}}$ and $\overline{\Gamma\mathrm{M}}$ azimuth in Supplementary Figure 2.

Finally, we note that the current observation of long-range repulsive interactions does not exclude the possibility of short-range attractive interactions and it is the implementation in the KMC that reproduces the feature in the experimental data. Short-range interactions may rather occur within a length scale that corresponds to intra-cell diffusion[45] while the discrete grid in terms of the KMC simulations allows just for interactions at the the intercell diffusion length-scale to be taken care of.

Supplementary References

1. Tamtögl, A. *et al.* Graphene on Ni(111): Electronic Corrugation and Dynamics from Helium Atom Scattering. *J. Phys. Chem. C* **119**, 25983–25990 (2015).
2. McMillan, J. A. & Los, S. C. Vitreous Ice: Irreversible Transformations During Warm-Up. *Nature* **206**, 806–807 (1965).
3. Löfgren, P., Ahlström, P., Lausma, J., Kasemo, B. & Chakarov, D. Crystallization Kinetics of Thin Amorphous Water Films on Surfaces. *Langmuir* **19**, 265–274 (2003).
4. Chakarov, D. V., Oesterlund, L. & Kasemo, B. Water adsorption and coadsorption with potassium on graphite(0001). *Langmuir* **11**, 1201–1214 (1995).
5. Chakarov, D., Österlund, L. & Kasemo, B. Water adsorption on graphite (0001). *Vacuum* **46**, 1109–1112 (1995).
6. Bolina, A. S., Wolff, A. J. & Brown, W. A. Reflection Absorption Infrared Spectroscopy and Temperature-Programmed Desorption Studies of the Adsorption and Desorption of Amorphous and Crystalline Water on a Graphite Surface. *J. Phys. Chem. B* **109**, 16836–16845 (2005).
7. Smith, R. S., Matthiesen, J. & Kay, B. D. Desorption kinetics of methanol, ethanol, and water from graphene. *J. Phys. Chem. A* **118**, 8242–8250 (2014).
8. Ward, D. J. *A Study of Spin-echo Lineshapes in Helium Atom Scattering from Adsorbates*. Ph.D. thesis, University of Cambridge (2013).
9. Poelsema, B. & Comsa, G. *Scattering of Thermal Energy Atoms*, vol. 115 of *Springer Tracts in Modern Physics* (Springer Berlin Heidelberg, 1989).

10. Glebov, A., Graham, A. & Menzel, A. Vibrational spectroscopy of water molecules on Pt(111) at submonolayer coverages. *Surf. Sci.* **427–428**, 22–26 (1999).
11. Ulbricht, H., Zacharia, R., Cindir, N. & Hertel, T. Thermal desorption of gases and solvents from graphite and carbon nanotube surfaces. *Carbon* **44**, 2931–2942 (2006).
12. Böttcher, S. *et al.* Graphene on ferromagnetic surfaces and its functionalization with water and ammonia. *Nanoscale Res. Lett.* **6**, 1–7 (2011).
13. Li, X. *et al.* Influence of water on the electronic structure of metal-supported graphene: Insights from van der Waals density functional theory. *Phys. Rev. B* **85**, 085425 (2012).
14. Ma, J. *et al.* Adsorption and diffusion of water on graphene from first principles. *Phys. Rev. B* **84**, 033402 (2011).
15. Leenaerts, O., Partoens, B. & Peeters, F. M. Water on graphene: Hydrophobicity and dipole moment using density functional theory. *Phys. Rev. B* **79**, 235440 (2009).
16. Ambrosetti, A. & Silvestrelli, P. L. Adsorption of Rare-Gas Atoms and Water on Graphite and Graphene by van der Waals-Corrected Density Functional Theory. *J. Phys. Chem. C* **115**, 3695–3702 (2011).
17. Freitas, R. R. Q., Rivelino, R., Mota, F. d. B. & de Castilho, C. M. C. DFT Studies of the Interactions of a Graphene Layer with Small Water Aggregates. *J. Phys. Chem. A* **115**, 12348–12356 (2011).
18. Wehling, T. O., Lichtenstein, A. I. & Katsnelson, M. I. First-principles studies of water adsorption on graphene: The role of the substrate. *Appl. Phys. Lett.* **93**, 202110 (2008).
19. Hamada, I. Adsorption of water on graphene: A van der Waals density functional study. *Phys. Rev. B* **86**, 195436 (2012).
20. Bertram, C., Fang, W., Pedevilla, P., Michaelides, A. & Morgenstern, K. Anomalously Low Barrier for Water Dimer Diffusion on Cu(111). *Nano Lett.* **19**, 3049–3056 (2019).
21. Tamtögl, A. *et al.* Nanoscopic diffusion of water on a topological insulator. *Nat. Commun.* **11**, 278 (2020).
22. Heidorn, S.-C., Bertram, C., Cabrera-Sanfelix, P. & Morgenstern, K. Consecutive Mechanism in the Diffusion of D_2O on a NaCl(100) Bilayer. *ACS Nano* **9**, 3572–3578 (2015).
23. Tocci, G., Joly, L. & Michaelides, A. Friction of Water on Graphene and Hexagonal Boron Nitride from *Ab Initio* Methods: Very Different Slippage Despite Very Similar Interface Structures. *Nano Lett.* **14**, 6872–6877 (2014).
24. Park, J. H. & Aluru, N. R. Ordering-Induced Fast Diffusion of Nanoscale Water Film on Graphene. *J. Phys. Chem. C* **114**, 2595–2599 (2010).
25. Ma, M., Tocci, G., Michaelides, A. & Aeppli, G. Fast diffusion of water nanodroplets on graphene. *Nat. Mater.* **15**, 66–71 (2015).
26. Perakis, F. *et al.* Diffusive dynamics during the high-to-low density transition in amorphous ice. *Proc. Natl. Acad. Sci.* (2017).
27. Xu, Y., Petrik, N. G., Smith, R. S., Kay, B. D. & Kimmel, G. A. Growth rate of crystalline ice and the diffusivity of supercooled water from 126 to 262 K. *Proc. Natl. Acad. Sci.* **113**, 14921–14925 (2016).
28. Foglia, F. *et al.* Aquaporin-like water transport in nanoporous crystalline layered carbon nitride. *Sci. Adv.* **6**, eabb6011 (2020).
29. Andersson, P. U., Suter, M. T., Marković, N. & Pettersson, J. B. C. Water Condensation on Graphite Studied by Elastic Helium Scattering and Molecular Dynamics Simulations. *J. Phys. Chem. C* **111**, 15258–15266 (2007).
30. Shih, C.-J., Strano, M. S. & Blankschtein, D. Wetting translucency of graphene. *Nat. Mater.* **12**, 866–869 (2013).
31. Standop, S., Michely, T. & Busse, C. H_2O on Graphene/Ir(111): A Periodic Array of Frozen Droplets. *J. Phys. Chem. C* **119**, 1418–1423 (2015).
32. Souda, R. & Aizawa, T. Crystallization kinetics of water on graphite. *Phys. Chem. Chem. Phys.* **20**, 21856–21863 (2018).
33. Henß, A.-K. *et al.* Density fluctuations as door-opener for diffusion on crowded surfaces. *Science* **363**, 715 (2019).
34. Wei, J. *et al.* The Dynamic Nature of CO Adlayers on Pt(111) Electrodes. *Angew. Chem. Int. Ed.* **59**, 6182–6186 (2020).
35. Sakong, S., Henß, A.-K., Wintterlin, J. & Groß, A. Diffusion on a Crowded Surface: kMC Simulations. *J. Phys. Chem. C* **124**, 15216–15224 (2020).
36. Alexandrowicz, G., Jardine, A. P., Hedgeland, H., Allison, W. & Ellis, J. Onset of 3D Collective Surface Diffusion in the Presence of Lateral Interactions: $\mathrm{Na/Cu(001)}$. *Phys. Rev. Lett.* **97**, 156103 (2006).
37. Alexandrowicz, G. *et al.* Observation of Uncorrelated Microscopic Motion in a Strongly Interacting Adsorbate System. *J. Am. Chem. Soc.* **130**, 6789–6794 (2008).
38. Ward, D. J. *et al.* Inter-adsorbate forces and coherent scattering in helium spin-echo experiments. *Phys. Chem. Chem. Phys.* **23**, 7799–7805 (2021).
39. Pusey, P. N. The dynamics of interacting Brownian particles. *J. Phys. A* **8**, 1433–1440 (1975).
40. Leitner, M., Sepiol, B., Stadler, L.-M., Pfau, B. & Vogl, G. Atomic diffusion studied with coherent X-rays. *Nat. Mater.* **8**, 717–720 (2009).
41. Sinha, S. & Ross, D. Self-consistent density response function method for dynamics of light interstitials in crystals. *Physica B+C* **149**, 51–56 (1988).
42. Jardine, A., Hedgeland, H., Alexandrowicz, G., Allison, W. & Ellis, J. Helium-3 spin-echo: Principles and application to dynamics at surfaces. *Prog. Surf. Sci.* **84**, 323 (2009).
43. Ala-Nissila, T., Ferrando, R. & Ying, S. C. Collective and single particle diffusion on surfaces. *Adv. Phys.* **51**, 949–1078 (2002).
44. Serra, A. & Ferrando, R. An efficient method for computing collective diffusion in a strongly interacting lattice gas. *Surf. Sci.* **515**, 588–596 (2002).
45. Stradner, A. *et al.* Equilibrium cluster formation in concentrated protein solutions and colloids. *Nature* **432**, 492–495 (2004).
